# Moderate evidence for the sex‐dependent effect of poisoning on adult survival in a long‐lived raptor species

**DOI:** 10.1002/ece3.70295

**Published:** 2024-09-18

**Authors:** Bernadett Zsinka, Szilvia Pásztory‐Kovács, Szilvia Kövér, Nóra Vili, Márton Horváth

**Affiliations:** ^1^ Department of Zoology University of Veterinary Medicine Budapest Budapest Hungary; ^2^ MME BirdLife Hungary Budapest Hungary

**Keywords:** bird of prey, conservation genetics, eastern imperial eagle, mark‐recapture, non‐invasive sampling

## Abstract

Survival rate is usually the greatest contributor to population growth in long‐lived species, and its accurate estimation along with the evaluation of the factors influencing it is therefore essential for effective conservation. Here, we studied the survival of breeding eastern imperial eagles *Aquila heliaca* in Hungary between 2011 and 2022 and investigated the effect of poisoning, the leading known anthropogenic cause of mortality. We used the Cormack‐Jolly‐Seber mark‐recapture model to estimate annual apparent survival and encounter probabilities based on the capture histories of 208 males and 411 females. We obtained encounter data from the DNA profiles of shed feathers collected at the nest sites, which we also supplemented with presences inferred from parentage analysis. The most supported model estimated a constant 91.6% annual survival over the study period, but models including the effect of sex and poisoning rate on survival had similar support. Sex difference in survival was less than 1% on average, but the survival of males decreased more with poisoning rate than the survival of females. However, due to smaller encounter probabilities, the estimates for males were less precise compared to females. Males may be more at risk from poisoning than females not only due to their more active foraging behaviour during incubation and chick‐rearing but also due to their smaller body size. Apart from providing direct practical information for the conservation management of imperial eagles, our results also highlight the importance of long‐term studies for estimating population parameters of birds of prey.

## Graphical abstract

Survival estimation of adult imperial eagles revealed that poisoning activity may have a stronger negative effect on the survival of the smaller‐bodied, more actively foraging males than on females. The genetic‐based mark‐recapture analysis was conducted on data of over 600 birds breeding in Hungary between 2011 and 2022.

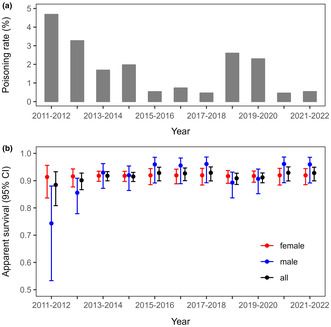

## 1 INTRODUCTION

Survival is one of the key life‐history traits affecting the population growth rate (Begon et al., 1996) and, consequently, the viability of a population (Sibly & Hone, 2002). Therefore, the accurate estimation of age‐, life‐stage‐ or sex‐specific survival rates is of great importance in understanding the dynamics of a population (Hernández‐Matías et al., 2011; Heuck et al., 2020). In addition, assessing the changes in these demographic parameters in response to certain human pressures, such as habitat alteration, electrocution or persecution, can facilitate the effective conservation of endangered species (Demerdzhiev et al., 2015; López‐López et al., 2011; Selwood et al., 2015; Sergio et al., 2021; Whitfield et al., 2004).

In the case of raptors (Accipitriformes, Falconiformes, Cathartiformes and Strigiformes), survival rate is a critical determinant of population growth: since raptors are generally long‐lived species with late maturity and low fecundity rates, their population growth rates are more sensitive to survival than to reproductive parameters (Katzner et al., 2006; Newton et al., 2016; Sæther & Bakke, 2000; Sergio et al., 2011; Stahl & Oli, 2006). Therefore, studies on raptor survival are especially important, considering that approximately 20% of raptor species are threatened, and more than half of all raptor species have a decreasing global population (McClure et al., 2018).

One of the major threats to raptors is poisoning, which can be intentional (directly aimed at controlling predator species) or accidental (resulting from the misuse of pesticides/rodenticides or from secondary poisoning through consumed poisoned prey) (McClure et al., 2018). Even though poisoning is known to have detrimental effects on populations (Köhler & Triebskorn, 2013), direct evidence linking poisoning to increased mortality and population decline remains scarce (Hernández & Margalida, 2009; Mateo‐Tomás et al., 2020; Ortega et al., 2009; Tenan et al., 2012; Whitfield et al., 2004).

Sex differences in survival are generally small in raptors (Hernández‐Matías et al., 2011; Hunt et al., 2017; Morrison, 2003; Newton et al., 2016), with either males or females having lower survival rates, and the direction of difference can be inconsistent even within a species (Dobson, 1987; Newton et al., 1983). Sex differences in survival rates are considered to be driven mainly by differences in selective forces on life‐history traits resulting in different trade‐off optima between survival and reproduction (dimorphic parental behaviour, defence of territory and body size), while the genetic hypotheses (unguarded X, mother's curse) seem to be less important (Connallon et al., 2022; Maklakov & Lummaa, 2013; Sumasgutner et al., 2019). In raptors, females tend to be larger and spend more time at the nest during the breeding season, while the males are responsible for providing food for their mates and chicks and defending the territory (Newton, 1979; Warkentin et al., 2016). These sex differences may lead to different survival rates in various ways. For example, the larger size of females could promote not only a higher chance of survival during the winter (Payevsky, 2016) but also a larger risk of electrocution (Ferrer & Hiraldo, 1992). Furthermore, the more sedentary behaviour of females during the breeding season could either result in less exposure to anthropogenic mortality factors (Hernández‐Matías et al., 2011) or a higher risk of mortality if they are targeted at the nest (Ewing et al., 2023).

Estimating the survival of raptors can be challenging because they appear in generally low densities and are often difficult to capture (Newton et al., 2016). Hence, the population dynamics of most raptor species are still poorly understood (Buechley et al., 2019; McClure et al., 2022). Survival is often estimated using mark‐recapture models, which require marking individuals and monitoring their fate through live encounters or dead recoveries (Lebreton et al., 1992; Sandercock, 2020). Conventional marking techniques (e.g. rings, wing tags, transmitters) all require the capture of birds, which is often not feasible for breeding adults, especially for large‐sized species. In such cases, DNA profiling from non‐invasively collected samples (e.g. shed feathers from the nest site) can provide an alternative method for identifying and monitoring individuals (Gil‐Sánchez et al., 2021; Horváth et al., 2005; Kenward et al., 2007; Rudnick et al., 2005; Vili, Szabó, et al., 2013). Another genetic‐based identification used to estimate the turnover of breeding individuals utilises DNA samples from chicks (Kylmänen et al., 2023; Ponnikas et al., 2017). In this case, the turnover of breeders is estimated based on relatedness between chicks of the same nest from consecutive years. While the plucked feathers and blood obtained from chicks are considered more reliable sources of DNA than shed feathers (Bush et al., 2005), this technique requires a high‐resolution marker set for reliable relatedness estimation.

Here, we investigate the survival of breeding individuals in the eastern imperial eagle *Aquila heliaca* (hereafter ‘imperial eagle’). As a Vulnerable (BirdLife International, 2019), large‐sized, long‐lived raptor, the survival rate of breeding individuals is a particularly important demographic parameter for the species. The imperial eagle's distribution is scattered throughout the Palearctic region, with only a few thousand breeding pairs worldwide (BirdLife International, 2019). Its westernmost population is found in the Pannonian Region (Demerdzhiev, Horváth, et al., 2011; Horváth et al., 2002) and consists of ca. 356–381 nesting pairs as of 2019 (Karyakin, 2020), making it the largest unified population outside of Russia and Kazakhstan (Horváth et al., 2011). Most of these Pannonian population (287 pairs as of 2019) reside in Hungary (Horváth et al., 2020). Imperial eagles exhibit floater behaviour in their first years and usually start breeding in their third or fourth calendar year (Horváth, 2022). The breeding success of these immature birds is lower than that of adults (Horváth et al., 2014). Following 1–2 years of successful breeding, adult breeders display high territory and mate fidelity (Rudnick et al., 2005; Vili, Szabó, et al., 2013). Sexual dimorphism in eastern imperial eagles only involves differences in size, with females being the larger sex, and behavioural dimorphism during the breeding season. Similarly to its sister species, the Spanish imperial eagle *Aquila adalberti* (Margalida, González, Sánchez, Oria, & Prada, 2007), eastern imperial eagle males also generally spend less time around the nest than females since they rarely take part in incubation, and they play the major role in food provisioning during the chick‐rearing period (Dobrev, 2009; Horváth, 2022). The imperial eagle is threatened by various anthropogenic factors, such as habitat fragmentation and alteration (Demerdzhiev et al., 2022), electrocution and persecution, including the illegal poisoning and shooting of birds (Deák, Fatér, et al., 2020; Lazarova et al., 2020). Even though the most dangerous pesticides (e.g. carbofuran) were banned in the EU in 2008, poisoning cases due to these substances still occur in imperial eagles, as well as in several other raptors (Deák, Árvay, & Horváth, 2020; Kitowski et al., 2020). In Hungary, poisoning was the leading known cause of mortality between 2005 and 2019, representing 25%–35% of all detected mortality cases (Deák, Fatér, et al., 2020; Horváth et al., 2011). Most of these were the result of intentional poisoning incidents, where baits (such as chicken eggs, carcasses of smaller prey animals or parts of large animals) poisoned with legally banned pesticides or insecticides were deployed with the aim of eliminating avian or mammal predators (Deák, Árvay, & Horváth, 2020). The other cases resulted from accidental poisoning, which occurs from the misuse of chemicals aimed to control agricultural pests or rodents. These were usually the result of improperly installed bait stations and/or the use of banned pesticides, which raptors encounter by consuming poisoned prey animals or their carcasses (Deák, Árvay, & Horváth, 2020; Deák, Fatér, et al., 2020).

We used a mark‐recapture method based on genetic identification to estimate annual apparent survival and encounter probabilities for breeding imperial eagles in East Hungary between 2011 and 2022. We aimed to explore possible sex differences in survival and investigate the relationship between poisoning and the annual survival probabilities of breeding males and females. Due to the aforementioned behavioural dimorphism, we expected males to have lower survival rates than females, as they spend more time away from the nest during breeding and, consequently, were assumed to have a higher risk of encountering anthropogenic mortality factors. Additionally, we hypothesised that the effect of poisoning on survival will be sex‐dependent due to the seasonal patterns of this behavioural dimorphism and that of poisoning activity. Most cases of poisoning during the study years occurred in the first half of the year, with a peak in early spring (February–April), which coincides with the egg‐laying and incubation period (March–May) of the imperial eagle (Deák, Árvay, & Horváth, 2020; Dobrev, 2009; Horváth, 2022). Since this is the period when females rarely leave the nest and only males hunt for food, we expected males to be more exposed to poisoning than females. Besides, males are also significantly smaller in body size than females (even by 20%–25% in weight); therefore, the same amount of a poisonous chemical in a prey or bait could be more detrimental or even fatal for males. Since breeding imperial eagles are difficult to capture and mark with conventional methods, we used DNA profiles obtained from shed feathers for the individual identification of breeders. This method has been previously applied with success in two studies estimating survival in imperial eagles (Rudnick et al., 2005; Vili, Szabó, et al., 2013). In addition, we also utilised the long‐term genetic monogamy of the species to obtain additional presence data of breeding birds through parentage analysis (Rudnick et al., 2005).

## 2 MATERIALS AND METHODS

### 2.1 Sample collection

**FIGURE 1:**
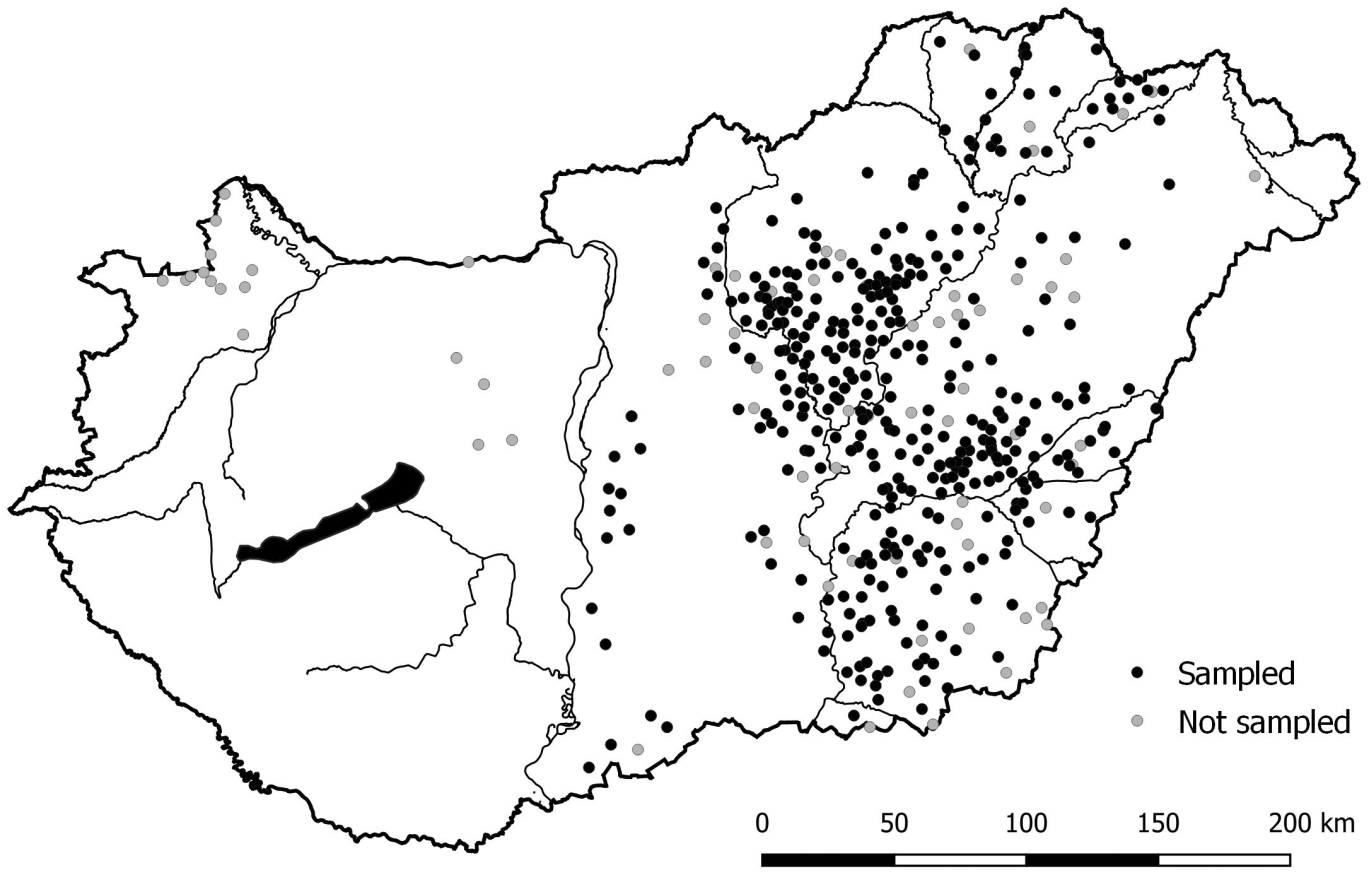
Distribution of the eastern imperial eagle in Hungary between 2011 and 2022 with sampled (*n* = 369, black) and not sampled (*n* = 73, grey) nests.

DNA samples for genetic profiling were collected between June and September each year between 2011 and 2022 in East Hungary in the frame of the national monitoring scheme for the imperial eagle in Hungary (Figure 1). The eastern part of the country holds about 95% of the national population of imperial eagles. On average, 67% of the nests here were sampled yearly (SD: 13%), with the highest coverage in 2017 (86%, 191 nests) and the lowest in 2012 (51%, 77 nests). Armpit feathers were plucked from nestlings during ringing, and breeding individuals were non‐invasively sampled by collecting their shed feathers around the 100 m radius of the nest. Plucked feathers were stored at −20°C in 2 mL microtubes filled with 96% ethanol, while shed feathers were stored in tagged plastic bags in dark, dry, and cool conditions to preserve DNA (Vili, Nemesházi, et al., 2013). We processed the majority (90%) of the samples within 1 year and all samples within 3 years of collection.

### 2.2 DNA extraction

We extracted the whole‐genome DNA using the Omega E.Z.N.A.® Tissue DNA Kit (Omega Bio‐tek Inc.) following the manufacturer's instructions but using an additional 20 μL of dithiotreitol (1 M) to aid in the digestion of keratin (Weigmann, 1968). We extracted DNA from the tip of the calamus in plucked nestling feathers and from the superior umbilicus region of shed feathers (Horváth et al., 2005).

### 2.3 Molecular sexing

We conducted molecular sexing on each feather by amplifying introns of the sex chromosome‐linked CHD1 gene, using the primers CHD‐i16F/CHD‐i16R (Suh et al., 2011). The PCR reaction included 0.065 μL DreamTaq polymerase (Fermentas), 1.7 μL 10X DreamTaq Green Buffer (Fermentas), 0.65 μL 25 mM MgCl_2_ (Thermo Scientific), 0.65 μL 2 mM dNTP mix (Thermo Scientific), 1–1 μL 10 pmol/μl forward and reverse primers, 8 μL H_2_O and 4 μL ca. 50 ng/μl concentration DNA. The PCR programme for molecular sexing constituted of an initial denaturation step at 95°C for 2 min, a touchdown section of nine cycles (denaturation: 95°C for 30s, annelation: temperature lowering by 1°C each cycle from 60 to 52°C and lasting 45 s, elongation: 72°C for 45 s), followed by 28 cycles of 95°C for 30s, 52°C for 45 s and 72°C for 45 s, ending with a final elongation of 7 min at 72°C. The PCR products were visualised through gel electrophoresis by UV illumination (2% agarose gel stained with EcoSafe (Pacific Image Electronics Co., Ltd) intercalator, 100 V, 45 min); the heterogametic females display two bands while the homogametic males only one.

### 2.4 Individual genotyping

We used nine microsatellite markers for individual identification. Out of these, two tetranucleotide loci (IEAAAG09 and IEAAAG11) (Busch et al., 2005) were optimised for the imperial eagle, five dinucleotide loci (Aa02, Aa35, Aa36, Aa39 and Aa43) (Martínez‐Cruz et al., 2002) were published for the Spanish imperial eagle *Aquila adalberti* and two dinucleotide loci (Hal04 and Hal10) (Hailer et al., 2005) for the white‐tailed eagle *Haliaeetus albicilla*. The 5′ end of the forward primers was modified with the following fluorescent dyes (Applied Biosystems™): 6‐FAM™ for Aa02, Aa39, Aa43, IEAAAG09, IEAAAG11; HEX™ for Aa35 and Aa36; and NED™ for Hal04 and Hal10. The 5′ end of the reverse primer was modified with a pigtail 5′GTTT sequence in the case of Aa02, Aa36 and Aa39.

We performed PCR reactions in a 10 μL volume, containing 2 μL 5xFIREPol® Master Mix (Solis BioDyne), which consists of dNTP mix, MgCl_2_ and Taq DNA‐polymerase, 0.5–0.5 μL 10 pmol/μl forward and reverse primers, 5 μL H_2_O and 2 μL ca. 50 ng/μl concentration DNA. Aa36 and Aa39, Aa35 and Aa43, Hal04 and Hal10, IEAAAG09 and IEAAAG11 were also amplifiable as duplexes. We used the PCR procedure described in Martínez‐Cruz et al. (2002) for all Aa loci and applied a modified version of it (touchdown scheme: 66–60°C, annealing at 60°C for 31 cycles) to the IEAAAG loci. For the Hal loci, we used the PCR profile described in Hailer et al. (2005), with the following modifications: 37 cycles, 45 s for both annealing and amplification. Fragment lengths were determined using capillary electrophoresis: PCR products were run on an ABI3130 sequencer (Applied Biosystems, using Gene Scan™ ‐500LIZ™ Size Standard), and we identified and scored alleles with OSIRIS v2.16 (Goor et al., 2011). We performed the fragment analysis similarly to the suggestions of Beja‐Pereria et al. (2009) by scoring each sample three times independently. Genotypes were assigned blind to the origin of the sample. We checked the deviation from the Hardy–Weinberg equilibrium with Genepop v1.2.2. (Rousset, 2008) and investigated the possible occurrence of null alleles and allelic dropouts using microchecker v.2.2.2 (Van Oosterhout et al., 2004). We calculated probabilities of identity (PI and PI_SIB_) (Waits et al., 2001) and exclusion probabilities for parentage analysis (P1X and P2X) with GenAlEx v.6.503 (Peakall & Smouse, 2006).

### 2.5 Constructing capture histories

We constructed capture histories (yearly presence–absence data for each individual) from two types of presences: (i) direct presences, when a breeding bird was sampled and genetically profiled directly from its shed feathers and (ii) indirect presences, when the presence of a breeding bird was inferred through parentage analysis due to the lack of shed feathers in that specific year. We could only obtain indirect presences for an individual if both the profile of its mate and at least one of its chicks were known from that specific year and the individual in question had also been profiled in another year (preceding or following the year in focus). We performed parentage analyses for indirect presences manually.

### 2.6 Survival analysis

We conducted the survival analysis in the MARK v9.0 software (White & Burnham, 1999) using the RMark v3.0.0 R interface (Laake, 2013) by fitting the Cormack‐Jolly‐Seber (CJS) open population model (Amstrup et al., 2006) to the capture histories. The CJS model estimates annual apparent survival probabilities (ϕ) for each one‐year interval and encounter probabilities (*p*) for each year as parameters of a generalised linear model. As sampling took place in June each year, these apparent survival probabilities refer to one‐year intervals. The estimated survival probability is apparent by definition, as it is the joint probability of surviving and remaining in the sampling area. However, since imperial eagles display high territory fidelity between years (Rudnick et al., 2005; Vili, Szabó, et al., 2013), we considered the apparent survival as a good proxy for survival in this case. Therefore, we will refer to ‘apparent survival’ as ‘survival’ throughout the article.

We constructed a candidate model set based on previous knowledge of the factors potentially influencing survival and encounter probabilities in this species. Both parameters were assumed to depend on sex (*sex*) based on the difference in behaviour the sexes display during breeding. We expected males to have lower survival and encounter probabilities than females, because we assumed that their more active foraging behaviour during the breeding results in a higher risk of mortality and a lesser probability of finding their shed feathers at the nest site. We investigated annual variation in survival and encounter probabilities with time‐dependency (*time*) models. We also assumed that survival probability would show variation across the years not only because of annual variation, for example, in food supply or weather conditions, but also due to varying levels of poisoning in the last decade. The number of poisoning incidents was taken from the BirdCrime Database of MME BirdLife Hungary, which incorporates all detected cases by the conservation organisations of the country (Deák, Árvay, & Horváth, 2020). To investigate the relationship between survival and poisoning, we modelled survival as a function of annual poisoning rate (*poison*), which we calculated as follows: number of poisoned imperial eagles found in East Hungary in each interval (e.g. between 1 June 2011 and the 31 May 2012)/total number of known nesting individuals (twice the number of known nesting pairs) at the beginning of the interval, multiplied by 100 to be expressed as a percentage. Therefore, a 1% poisoning rate means that the observed number of poisoned imperial eagles equals 1% of the known nesting individuals. We presumed that the number of known nesting pairs accounts for at least 95% of the total number of nesting pairs in East Hungary (Horváth et al., 2011). We emphasise that the number of poisoned imperial eagles includes not only nesting individuals but also floaters as well as individuals of unknown age. That is because determining whether the poisoned bird was a breeder or a floater was not possible in a high number of cases. First, there was no available information on age for 34% of the poisoned birds. Second, since imperial eagles can start breeding as early as three calendar years (Horváth, 2022), poisoned birds with immature colouration (10%) could have been either floaters or breeders. Only birds in their first or second calendar year (30%) could have been safely excluded as non‐breeders. However, due to the high number of poisoned birds with unknown age or unsure status, we decided to include all poisoned individuals when calculating poisoning rates instead of discarding a significant number of detected cases. Therefore, the poisoning rate used here is only a proxy for the true poisoning rate of breeding individuals. Assuming that the observed poisoning rate of all birds is directly proportional to the poisoning rate of breeding birds, yearly changes in this observed poisoning rate reflect the yearly changes in the true poisoning rate of breeding birds. Since August 2013, a poison and carcass detection dog (PCDD) unit has also been used to help uncover poisoning incidents and proved to be more successful in both carcass and bait detection than human investigators (Deák, Árvay, & Horváth, 2020). Therefore, we corrected the number of poisoned carcasses found before August 2013 for the possibly undetected poisoning events to gain a more accurate estimation of poisoning rates in these early intervals. We did this by assuming the detection probability by the PCDD unit to be 1 and then dividing the number of poisoned imperial eagle carcasses found before August 2013 by the detection probability of human investigators. This probability was estimated to be 0.81 since out of the 42 imperial eagle carcasses detected between August 2013 and August 2020, 34 were recovered by human investigators (Deák, Árvay, & Horváth, 2020; Deák's personal communication). Lastly, we also modelled encounter probabilities as a function of sampling effort (*effort*), which we calculated for each year as the number of genotyped feathers (shed and chick feathers for parentage analysis included)/total number of known nesting individuals. Controlling for the number of nesting pairs in the case of both *poison* and *effort* was necessary due to the rapid expansion of the population throughout the study (Horváth, 2022).

We used the standard CJS model {ϕ(*sex* × *time*), *p*(*sex* × *time*)} as the general model, and the candidate model set included the variables mentioned above with both additive (+) and interaction terms (×). We also considered models with constant (.) survival or encounter probability. We constructed the *sex* models with males as the reference category.

Assumptions of the CJS model include the homogeneity of survival and encounter probabilities among groups of marked individuals (Amstrup et al., 2006). Therefore, we used goodness‐of‐fit tests to assess whether these conditions are met in our dataset. We investigated the goodness‐of‐fit of our general model to the capture histories with the $\chi^{2}$ tests of programme RELEASE v.3.0 (Burnham et al., 1987). We estimated the overdispersion parameter ($\hat{c}$) by dividing the overall $\chi^{2}$ of the component tests by the overall degrees of freedom. Then, we used this $\hat{c}$ estimate to adjust the AIC_c_ (Akaike Information Criterion corrected for small sample size) values of models, resulting in a QAIC_c_‐based model selection. We also adjusted AIC_c_ values by matching the parameter counts (*K*) to the model structure and by considering confounded parameters, as recommended by Cooch and White (2014, Chapter 4). We ranked the models using their QAIC_c_ values and considered them equally parsimonious if the difference in their QAIC_c_ values (∆QAIC_c_) was less than two (Burnham & Anderson, 2002). We used the Akaike weights (*w*_*i*_) to evaluate the relative support of the competing models.

We used R v.4.3.1. (R Core Team, 2023) to run RMark and calculate descriptive statistics of genotype data. To create figures, we used the ggplot2 v.3.4.4. R Package (Wickham, 2016) and QGIS v.3.28.1. (QGIS Development Team, 2023). Data and code are provided as supplementary files (S1).

## 3 RESULTS

### 3.1 Individual identification

During the study, we genotyped 1730 shed feathers, which belonged to 619 breeding individuals (208 males and 411 females) with an average of 2.8 samples per individual. Out of the 15,570 genotyped loci, 1172 (7.53%) failed to amplify adequately, and we detected 106 genotyping errors (0.74%) by comparing multiple samples of the same individual. In total, we found 58 alleles at the nine microsatellite loci, ranging from four (IEAAAG09) to ten (Aa35), with an average of 6.4 alleles per locus. PI and PI_SIB_ values for the complete marker set were 9.5 × 10^−9^ and 5.7 × 10^−4^, respectively. Exclusion probabilities P1X and P2X were 0.997 and 0.963, respectively. We found deviations from the Hardy–Weinberg equilibrium (*p* < .05) in three loci (Aa35, Aa36 and Aa43), with the possibility of null alleles on locus Aa36 (Tables S1 and S2). As null alleles do not amplify during PCR, their presence on a locus leads to falsely identifying heterozygotes as homozygotes, which can result in false mismatches during the comparison of true parent‐offspring genotypes, that is, falsely excluding breeders as parents of a chick. That is because the chick with the null allele appears to be homozygous, while it is expected to be heterozygous based on the putative parents' genotypes. To avoid such false exclusions, we allowed for such mismatches on locus Aa36 during parentage analysis if all other loci indicated a match between the breeder and the chick.

### 3.2 Survival analysis

Capture histories of the 208 males and 411 females consisted of a total of 1712 presences (436 presences for males and 1276 presences for females), out of which 181 were indirect presences, that is, encounter data obtained via parentage analysis (111 and 70 for males and females, respectively). 3% of the males and 20% of the females encountered on the first occasion were also encountered on the last occasion of the 12 years study (one male and 17 females).

Based on the RELEASE goodness‐of‐fit test results, our dataset satisfied the condition of homogeneous encounter probabilities for both males and females. However, we found some violations of the assumption of homogeneous survival probabilities for females (*p* = .023). This could be mainly attributed to the results of females encountered in 2012, 2014 and 2017. In all three cases, individuals encountered for the first time in the given year were indicated to have lower survival probabilities than those that had been encountered before. Overall, we detected overdispersion to be low, with an estimate of $\hat{c}$ = 1.148, and we carried out the model selection procedure after adjusting the AIC_c_ values with this $\hat{c}$ estimate.

In each of the four most supported models (∆QAIC_c_ < 2, Σ*w*_*i*_ = 0.746), the encounter probability was a function of *sex* × *time* (Table 1). Regarding survival, several different models received similar support, as detailed below.

**TABLE 1:** Model selection results for Cormack‐Jolly‐Seber models estimating annual apparent survival (ϕ) and encounter probability (*p*) as a function of sex, time and poisoning rate for breeding eastern imperial eagles in East Hungary, 2011–2022. Poisoning rate was calculated as the number of poisoned imperial eagles found/number of nesting individuals x 100.

Model	QAIC_c_	ΔQAIC_c_	*w*_*i*_	*K*	Deviance
{ϕ(.), *p*(*sex* × *time*)}	3250.20	0.00	0.268	23	1042.56
{ϕ(*poison*), *p*(*sex* × *time*)}	3250.90	0.70	0.188	24	1041.19
{ϕ(*sex* × *poison*), *p*(*sex* × *time*)}	3250.92	0.72	0.187	26	1037.07
{ϕ(*sex*), *p*(*sex* × *time*)}	3252.09	1.89	0.104	24	1042.39
{ϕ(*sex + poison*), *p*(*sex* × *time*)}	3252.77	2.57	0.074	25	1041.00
{ϕ(.), *p*(*sex* + *time*)}	3253.11	2.91	0.062	13	1065.97
{ϕ(*poison*), *p*(*sex* + *time*)}	3254.15	3.96	0.037	14	1064.97
{ϕ(*sex*), *p*(*sex* + *time*)}	3254.38	4.18	0.033	14	1065.19
{ϕ(*sex* + *poison*), *p*(*sex* + *time*)}	3255.42	5.22	0.020	15	1064.19
{ϕ(*sex* × *poison*), *p*(*sex* + *time*)}	3256.21	6.00	0.013	16	1062.94
{ϕ(*sex* × *time*)*, p*(*sex* × *time*)}	3273.28	23.07	0.000	42	1025.93

*Note*: QAIC_c_: Quasi‐Akaike Information Criterion corrected for small sample size and adjusted with $\hat{c}$ = 1.148, ΔQAIC_c_: Difference in the QAIC_c_ values of the model and the best‐supported model, *w*_*i*_: Model weight, *K*: Model parameter count. Models receiving little support (*w*_*i*_ < 0.01) are not shown, except for the general model {ϕ(*sex* × *time*), *p*(*sex* × *time*)}.

The minimum QAIC_c_ model {ϕ(.), *p*(*sex* × *time*)} estimated survival as a constant probability over time with a 0.916 ± 0.008 SE probability of survival independent of sex. Models in which survival was a function of *poison, sex* × *poison*, and *sex* were equally parsimonious (∆QAIC_c_ < 2, Table 1).

**FIGURE 2:**
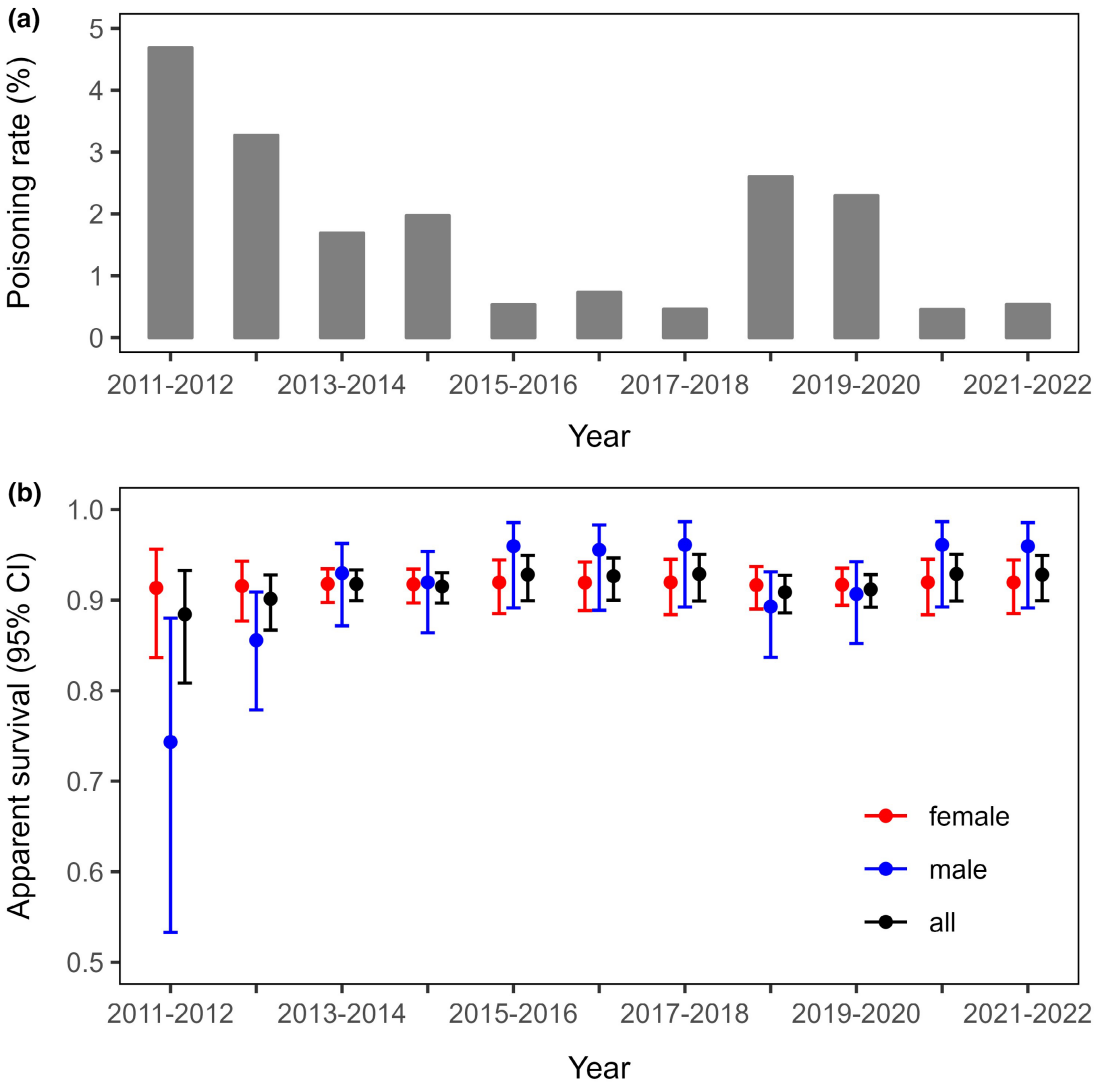
Poisoning rates (number of poisoned eastern imperial eagles found/number of nesting eastern imperial eagles × 100) in East Hungary between 2011 and 2022. Source: BirdCrime Database of MME BirdLife Hungary (a). Estimated apparent survival probabilities (ϕ± 95% CI) of breeding eastern imperial eagles from models {ϕ(*sex* × *poison*), *p*(*sex* × *time*)} (blue: Male, red: Female) and {ϕ (*poison*), *p*(*sex* × *time*)} (black: All) (b).

The poisoning rate (number of poisoned imperial eagles found/number of nesting individuals x 100) showed two peaks during the studied period, in intervals 2011–2012 and 2018–2019 (Figure 2a). Based on model {ϕ(*poison*), *p*(*sex* × *time*)}, poisoning had a negative relationship with survival, resulting in a 0.929 ± 0.013 SE estimated survival for the interval with the lowest poisoning rate (0.46%, 2017–2018) and a 0.884 ± 0.031 SE survival probability for the interval with the highest poisoning rate (4.68%, 2011–2012) (Figure 2b, black). However, the CI for the coefficient of the poisoning effect overlapped zero (*β* = −0.127, 95%CI = −0.333 to +0.080).

Males were estimated to have a lower survival probability than females when survival was assumed to be sex‐dependent but constant in time (model {ϕ(*sex*), *p*(*sex* × *time*)}). However, this difference was small, and the CI for the coefficient of the sex effect overlapped zero (males: 0.909 ± 0.018 SE, females: 0.918 ± 0.009 SE, *β* = +0.106, 95%CI = −0.392–0.605). This model received somewhat less support (*w*_*i*_ = 0.104) than model {ϕ(*sex* × *poison*), *p*(*sex* × *time*)} (*w*_*i*_ = 0.187), indicating that the difference in the survival of males and females may be attributed to their survival being affected by poisoning to a different degree. Based on the estimates from this model, only male survival probability was affected significantly by poisoning (*β* = −0.507, 95%CI = −0.927 to −0.087) (Figure 2b, blue), implying that poisoning may have a greater effect on the survival of males compared to females.

Regarding the unconstrained time model {ϕ(*sex* × *time*), *p*(*sex* × *time*)}, some of the survival parameters were not estimable (characterised by large SEs or SE = 0.000) due to the high number of parameters compared to the amount of data available. Those that were estimable were mostly in agreement with the estimates from the ϕ(*sex* × *poison*) model, as the estimated female survivals showed no clear relationship with poisoning and the male estimates were lower in the 2 years with the highest poisoning rates. The only interval when the estimates of the two models were clearly different is 2017–2018, when the unconstrained model estimated low male survival despite a low poisoning rate (Table S2).

**FIGURE 3:**
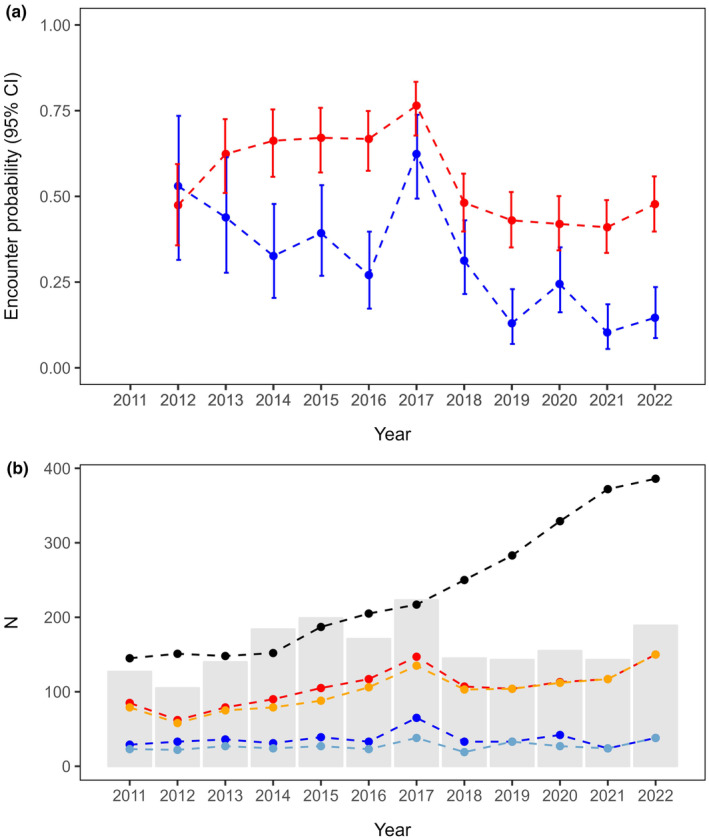
Estimates of yearly encounter probabilities (*p*) from model {ϕ(.), *p*(*sex* × *time*)} for breeding male (blue) and female (red) eastern imperial eagles in East Hungary, 2011–2022 (a). Sample sizes of the mark‐recapture analysis on breeding eastern imperial eagles in East Hungary, 2011–2022 (b): Number of nesting pairs (black), number of males identified (all presences: Dark blue; only direct presences from shed feathers: Light blue), number of females identified (all presences: Red; only direct presences from shed feathers: Orange) and number of feathers (shed and chick feathers) genotyped (grey bars).

Estimated encounter probabilities were lower for males than females in all models where encounter probability was a function of sex. Estimates of encounter probabilities from the best‐supported model {ϕ(.), *p*(*sex* × *time*)} ranged from 0.103 ± 0.032 SE (in 2021) to 0.624 ± 0.064 SE (in 2017) for males and from 0.410 ± 0.040 SE (in 2021) to 0.765 ± 0.040 SE (in 2017) for females (Figure 3a). This result generally coincides with the percentage of nesting individuals identified each year, which were lower for males compared to females in all years and showed variation across the years, ranging from 6.50% (in 2021) to 30.0% (in 2017) for males and from 31.5% (in 2021) to 67.7% (in 2017) for females (Figure 3b). Values of yearly sampling effort (number of genotyped feathers/number of nesting individuals) showed an average of 0.379 ± 0.136 SD and ranged from 0.192 (in 2021) to 0.605 (in 2015) (Figure 3b). Models where encounter probability was constrained as a function of this effort covariate, despite having fewer parameters, received substantially less support than fully time‐dependent models (Σ*w*_*time*_/Σ*w*_*effort*_ = 0.987/0.007 = 141), which implies that the number of genotyped feathers was not an adequate predictor of encounter probability.

## 4 DISCUSSION

We estimated annual apparent survival probabilities for breeding eastern imperial eagles in the East Hungarian population using a mark‐recapture method based on genetic identification. Although the estimated survival probabilities are only apparent by definition, as the CJS model does not separate actual survival and emigration from the sampling area (Amstrup et al., 2006), we presume that since breeding imperial eagles exhibit high territory fidelity (Rudnick et al., 2005; Vili, Szabó, et al., 2013), the estimated apparent survivals are good proxies to the actual survival probabilities. The previously reported high territory fidelity of breeding imperial eagles is also supported by our data, as over the 12 years of our study, only 9 males (4% of all males studied) and 44 females (9% of all females studied) were identified from more than one territory. Such encounters correspond to less than 4% of all the 1712 presences detected and, in most cases, birds moved only a short distance to the neighbouring territory, similarly to the observations of Vili, Szabó, et al. (2013).

The East Hungarian population we studied is the largest in the Western range of the imperial eagle's distribution, and therefore, investigating its demography plays a crucial role in preserving this species in Europe. Adult survival is often considered the most important demographic parameter for long‐lived species (Katzner et al., 2006; Sæther & Bakke, 2000; Stahl & Oli, 2006); thus, the results of this study are essential for a future viability analysis for this population (Lacy, 2019). Our estimates refer to the period 2011–2022, which was characterised by variable levels of poisoning and the intensive conservation of the species in the context of the Helicon LIFE (2012–2016, LIFE10NAT/HU/019) and PannonEagle LIFE (2017–2023, LIFE15 NAT/HU/000902) projects. We estimated >90% annual survival on average, a typical estimate for a large raptor such as the imperial eagle (Newton et al., 2016). However, our estimates were lower for the first years of the study when poisoning rates were higher. These results agreed with the pattern in the population growth curve of imperial eagles during this period: a plateau was detected in the number of nesting pairs between 2011 and 2014 instead of the exponential growth observed before and after this period (Horváth, 2022, also shown in Figure 3b), implying that the high poisoning rates in 2011–2013 had major effects on population growth. Moreover, our data also provided moderate evidence for the sex‐dependent effect of poisoning on adult survival. The estimated negative effect of poisoning on survival was stronger in males than in females. Therefore, poisoning may lead to a female‐biassed adult sex ratio (ASR) in this population. This result implies that due to the strict monogamy in this species (Rudnick et al., 2005), the number of males may be a limiting factor to the growth of this population. A female‐biassed ASR can also affect the evolution of reproductive traits like age‐to‐maturation (Ancona et al., 2020) and may result in increased competition and therefore elevated mortality in females, which in turn could have adverse effects on population viability (Kokko & Jennions, 2008; Székely et al., 2014). To our knowledge, no direct evidence of sex difference in age‐to‐maturation is available in the eastern imperial eagle, but observations of a higher number of immature males than females in the breeding populations suggest that males may reach maturity at an earlier age than females (Demerdzhiev, Gradev, et al., 2011; Demerdzhiev, Stoychev, et al., 2011, Horváth's personal communication). Similar observations were also made for the Spanish imperial eagle (Margalida et al., 2008; Margalida, González, Sánchez, Oria, & Prada, 2007). An earlier maturation of males could mitigate the negative effect of male‐biassed mortality on population growth. However, it is important to note that a higher proportion of immature males in the breeding population may not necessarily mean that males reach maturity sooner than females but could also be the outcome of male‐biassed adult mortality.

The annual survival of breeding imperial eagles was estimated to be 91.6% on average. This survival estimate is close to the one estimated for the Bulgarian population based on an integrated population model (92.4%, Demerdzhiev et al., 2015). However, previous turnover‐based survival estimates for the imperial eagle were considerably lower: 84% for the population in Kazakhstan (Rudnick et al., 2005) and a maximum of 72.3% annual survival rate for females in this East Hungarian population between 1997 and 2006, with no estimate for males (Vili, Szabó, et al., 2013). The 72.3% estimate of Vili, Szabó, et al. (2013) is exceptionally low even compared to estimates obtained for the imperial eagle's sister species, the Spanish imperial eagle (92.7%–95.3%, Ferrer & Calderón, 1990; 91,5%, Ortega et al., 2009) and for other *Aquila* species (Bonelli's eagle *Aquila fasciata*: 83.9%–96.1%, Real & Mañosa, 1997; 87.0%, Hernández‐Matías et al., 2011; golden eagle *Aquila chrysaetos*: 91.0%, Whitfield et al., 2004; 90.0%, Millsap et al., 2022; 90.5% Hunt et al., 2017; 93.0%, Crandall et al., 2019).

In the following, we discuss whether poisoning could explain the previously estimated exceptionally low breeder survival rate for the Hungarian population or if some other factors might be responsible. In Hungary, the first two carcasses of illegally poisoned imperial eagles were recovered in 2005, and poisoning has been considered the leading anthropogenic mortality cause of imperial eagles ever since (Horváth et al., 2011). However, we suppose that poisoning was also present in earlier years, as suggested by the two reported cases of alleged poisoning in 1980–2000 (Horváth et al., 2011). Only one poisoning rate estimate is available for the time range of the study of Vili, Szabó, et al. (2013). This poisoning rate calculated for 2005–2006 is only 2.74%, which is very low for the estimated female survival rate of 72.3%. Our models predicted much higher survival rates for this relatively low poisoning rate: a 91.6% annual survival for females from the ϕ(*sex* × *poison*) model and a 90.7% annual survival on average for both sexes from the ϕ(*poison*) model.

Reasons behind these seemingly contradicting results can include (1) actual poisoning rates may have been higher than detected for the previous period, with a more substantial effect on the survival of females than the one reported here; (2) other factors than poisoning may have contributed to this very low survival rate; or (3) methodological differences between the two studies.

First, many poisoning incidents could have remained uncovered between 1997 and 2006 since no organised poison searches were conducted at the time, and carcasses were rarely tested for poison compounds (Deák's personal communication). Therefore, actual poisoning rates may have been much higher than detected. This is also implied by the high poisoning rates observed in the following years when the monitoring of poisoning was already in focus after the recovery of two poisoned imperial eagles in 2005 (Horváth et al., 2011). In addition, the survival of females may react to poisoning detectably only above a specific rate. One plausible explanation is that the frequent turnover of males at higher levels of poisoning could introduce a striking effect on the survival of females. This hypothesis is based on studies conducted on other monogamous bird species, which revealed that mate change can lead to a lower apparent survival (Culina et al., 2015; Jankowiak et al., 2018), even in long‐lived species (Leach et al., 2020).

Second, factors other than poisoning may have also caused the previously low survival of females. The frequency of electrocution, the second most important mortality factor of imperial eagles, remained more or less constant over the years (Deák, Fatér, et al., 2020) and thus cannot explain the differences in survival. Another possible factor could be the change in habitat. During the 1970s, imperial eagles in Hungary resided almost exclusively in the North Hungarian Mountains (Bagyura et al., 2002). These lower‐quality mountainous habitats served as a refuge for the species since lower human population density and closed vegetation in these areas ensured a lower risk of persecution (Horváth et al., 2011). However, the population has expanded significantly over the decades (Horváth, 2022), and a larger proportion now resides in lowland areas. These lowland areas support higher fledging success due to better foraging opportunities than the previously occupied mountainous areas (Horváth et al., 2014) and may also promote higher survival in breeders.

Lastly, methodological differences may have also contributed to the difference between our estimate and that of Vili, Szabó, et al. (2013). The latter was based on turnover rate (annual % of breeding individuals replaced by new breeding individuals), which does not account for imperfect detectability, while mark‐recapture does. Consequently, breeding dispersal could have introduced a negative bias in the survival estimate of Vili, Szabó, et al. (2013). However, considering the high territory fidelity exhibited by this species (Rudnick et al., 2005; Vili, Szabó, et al., 2013), dispersal only is unlikely to have caused the large difference in the estimates. Additionally, the estimate of Vili, Szabó, et al. (2013) was based on four times fewer individuals than our study and, therefore, carries more uncertainty.

Since Vili, Szabó, et al. (2013) only gave estimates for females, our study is the first to explore sex differences in survival in this population of imperial eagles. Models featuring the sex‐dependence of survival indicated lower male survival compared to females mainly attributable to the lower survival probabilities of males in years of high poisoning, especially in the first interval. There was only one interval where male survival, in contrast to the low poisoning rate, was estimated to be low by the unconstrained time model. This contradiction may be explained by the imperfect detection of poisoning events or by the increase of another mortality factor in this interval.

A possible mortality factor that may show yearly fluctuations similarly to the poisoning rate and could, therefore, interact with the effect of poisoning on breeder survival, is food supply. Due to their body size dimorphism, food shortages may affect males and females differently (Payevsky, 2016). Therefore, prey availability may be enhancing or counteracting the possible sex‐dependent effect of poisoning. The latter might explain why models with the sex‐dependent effect of poisoning were not better supported.

Another reason why the model selection may not have strongly supported the relationship between poisoning and survival is that in most years of the study, only a relatively low level of poisoning was observed. The negative effect of poisoning on survival may only be prominent at higher rates of poisoning. This is also suggested by the estimated survival probabilities (Figure 2b), as in years with low poisoning rates, the CIs of male and female estimates largely overlap, and the sex difference in survival only appears to be relevant in the first intervals, when poisoning rates were much higher.

In contrast to our results of lower male survival, a previous study on imperial eagles implied higher survival for males in Kazakhstan (Rudnick et al., 2005) and in the case of the Spanish imperial eagle, both female‐biassed mortality (Ferrer & Hiraldo, 1992) and no difference in the survival of the sexes have been reported (Ortega et al., 2009). In raptors, sex‐dependent survival can be expected because of behavioural dimorphism (Newton et al., 2016), through which different environmental factors, including human persecution, influence the sexes differently (Ewing et al., 2023; Hernández‐Matías et al., 2011). Our results suggest this may also be true for the imperial eagle. In spring, females spend most of their time at the nest incubating and males are the ones foraging for food (Dobrev, 2009; Horváth, 2022). This period of primarily male foraging coincides with the reported annual peak of poisoning activity from February to April (Deák, Árvay, & Horváth, 2020). This was also true in the first interval of our study, when the difference between male and female survival was estimated to be the highest and 64% of the poisoned imperial eagles were recovered between the end of February and the end of April. We hypothesise that this temporal pattern of poisoning activity, coupled with the mentioned behavioural dimorphism, could explain why males may be more at risk from poisoning than females. Moreover, although males could bring back poisoned food to the nest for the females, their quick death after exposure to the poison might prevent them from doing so. During our study, the most commonly used pesticide for the intentional killing of raptors in Hungary was carbofuran (Deák, Árvay, & Horváth, 2020), which can lead to death in minutes following its ingestion (Lehel et al., 2010; López‐Bao & Mateo‐Tomás, 2022). Therefore, foraging males have only a slight chance of bringing poisoned baits back to the nest as they are very likely to die on site, explaining why they may fall victim to poisoning more frequently than their mates. To further investigate whether breeding males are more threatened by poisoning than females, assessing the age and sex of poisoned imperial eagles would be required.

This previously mentioned behavioural dimorphism also resulted in a smaller sample size and smaller encounter probabilities for males compared to females, since the shed feathers of males could be recovered with substantially less success around the nest. Due to this, our survival estimates for males are less precise than for females. This was especially true for 2011–2012, which was not only the first interval of the study but also had a much higher poisoning rate than the average. Both factors contributed to the fact that less data were available for the estimates for this period. Thus, the uncertainty of these estimates was greater than that of later years.

To obtain more encounter data, especially on males, we also used parentage analysis to infer the presence of breeders. Supplementing our dataset with these indirect presences was made possible by the high territory and mate fidelity of the species. However, this method could have introduced heterogeneity to encounter probabilities since indirect presences could only be obtained of those birds that had been identified previously at least once and whose mate was also identified in the year in question. Additionally, indirect presences could have also introduced upward bias to our survival estimates. This would be the case if the probability of obtaining an indirect presence of an individual was positively correlated with its survival. This can happen in two ways: (i) since indirect presences can only be obtained of birds that are identified in at least one other year, there is a higher chance to obtain indirect presences of individuals that live longer; and (ii) since indirect presences are determined through parentage analysis, more data could be obtained on those birds that successfully produced offspring over several years, meaning that if breeding success is positively correlated with survival probability (see for example Espie et al., 2004), the probability of obtaining an indirect presence was higher for birds with better survival. In both cases, birds with better survival would be overrepresented in our dataset. This would be especially true for males, considering that about 25% of the male encounter data were indirect presences. Nevertheless, we found no evidence of indirect presences violating the assumptions of the CJS model based on the goodness‐of‐fit tests: overdispersion was estimated to be of an acceptable value ($\hat{c}$ = 1.148), which was even smaller than the one estimated for the dataset without indirect presences ($\hat{c}$ = 1.200). Thus, indirect presences could serve as a valuable tool in cases when encounter data are otherwise scarce, a situation typical for raptors (Newton et al., 2016).

RELEASE goodness‐of‐fit tests revealed a slight violation of the assumption of homogeneous survival probabilities in the case of females, both with and without indirect presences: females identified for the first time in 2012, 2014 and 2017 were less likely to be encountered later than females that have been encountered before. Assuming that birds that are identified for the first time in a specific year are mainly young (3–4 calendar years) birds entering the breeding stage, an explanation for this pattern could be the lower apparent survival of younger breeders. Reasons behind this could include a lower actual survival for younger individuals or a lower territory fidelity, leading to their dispersal to another breeding site outside of our study area after unsuccessful breeding attempts (Catlin et al., 2005; Jiménez‐Franco et al., 2013). There are two options to investigate if the survival of younger breeders is lower compared to adults. The first is to apply a time‐since‐marking model, but it requires that all the birds are young when identified for the first time, which is not a valid assumption in this case. The second option is the age‐dependent model; however, we know the age of only a few birds in this study. Continued observation of breeding individuals and incorporation of ringing and satellite‐tracking data of young breeders could facilitate answering this question in the future.

Our study provided the first estimates of sex‐dependent survival for imperial eagles in Hungary, a population of high conservation value due to its large size and peripheric placement in the distribution of the species. We also investigated the relationship between survival and poisoning rates in imperial eagles for the first time. In conclusion, the annual survival probability of breeding imperial eagles in East Hungary is high despite current anthropogenic threats in the country. The 26% increase in the survival estimate compared to the period of 1997–2006 implies that the conservation measures have contributed to the increase in survival of breeding birds. However, we cannot exclude methodological differences as explanations for the difference between previous and current estimates. Furthermore, while we found no definite evidence for the effect of poisoning and sex on adult survival, our results alert that male survival might be lower and more sensitive to poisoning than female survival. If that is the case, it would be important from both a conservational and evolutionary perspective, as sex‐biassed adult mortality is considered the main driver of biassed adult sex ratios in birds, which in turn influence parental roles and mating competition and can affect population viability (Ancona et al., 2020; Kokko & Jennions, 2008; Székely et al., 2014). Furthermore, in a species, such as the imperial eagle, where food provisioning during breeding is mostly dependent on the male and experienced adults have better hunting capacity (Margalida et al., 2008), male‐biassed mortality can also have a direct impact on productivity. While the scarcity of male data makes estimates of male survival less precise than that of females, our method of indirectly assessing the presence of breeders through parentage analysis greatly contributed to the data on males. To obtain a deeper understanding of the demography of the population, more precise estimates of breeding male survival would be beneficial in the future. Furthermore, the survival rates of the floater age groups and the young breeders are unknown; a future task is to estimate these from ringing and satellite‐tracking data. Moreover, incorporating these results into a population viability analysis could further facilitate our understanding of the effect of poisoning on survival and its consequences on population growth in this protected, long‐lived raptor.

## AUTHOR CONTRIBUTIONS

**Bernadett Zsinka:** Conceptualization (equal); data curation (equal); formal analysis (equal); investigation (equal); methodology (equal); visualization (lead); writing – original draft (lead). **Szilvia Pásztory‐Kovács:** Conceptualization (equal); data curation (equal); formal analysis (equal); investigation (equal); methodology (equal); project administration (equal); resources (equal); supervision (equal); visualization (supporting); writing – original draft (supporting); writing – review and editing (equal). **Szilvia Kövér:** Conceptualization (equal); formal analysis (equal); methodology (equal); supervision (equal); visualization (supporting); writing – original draft (supporting); writing – review and editing (equal). **Nóra Vili:** Investigation (supporting); writing – review and editing (supporting). **Márton Horváth:** Conceptualization (equal); data curation (equal); funding acquisition (lead); investigation (equal); project administration (equal); resources (equal); supervision (equal); writing – review and editing (supporting).

## CONFLICT OF INTEREST STATEMENT

The authors declare that they have no conflict of interest.

## Supporting information


Data S1.

Tables S1–S2.

## Data Availability

Data and code are provided as supplementary files S1.
