# Supplementary material for: Moderate evidence for the sex‐dependent effect of poisoning on adult survival in a long‐lived raptor species
Source: Ecol Evol. 2024 Sep 18;14(9):e70295. doi: 10.1002/ece3.70295 (PMC11410563; doi:10.1002/ece3.70295)
Supplement: Supplementary file 2 — Tables S1–S2. [file ECE3-14-e70295-s002.docx]

**Appendix**

Table S1. Microsatellite marker set used for the individual identification of breeding eastern imperial eagles in East Hungary between 2011 and 2022. Na: number of alleles, Ho: observed heterozygosity, He: expected heterozygosity, p-value of the Hardy-Weinberg equilibrium test. We found deviations from the Hardy-Weinberg equilibrium in the case of loci Aa35, Aa36 and Aa43.

| **Locus** | **Source** | **Na** | **Ho** | **He** | **p-value** |
| --- | --- | --- | --- | --- | --- |
| Aa02 | Martínez-Cruz et al. 2002 | 6 | 0.8228 | 0.7674 | 0.2370 |
| Aa35 | Martínez-Cruz et al. 2002 | 10 | 0.8211 | 0.8184 | 0.0000 |
| Aa36 | Martínez-Cruz et al. 2002 | 6 | 0.7023 | 0.7653 | 0.0014 |
| Aa39 | Martínez-Cruz et al. 2002 | 9 | 0.7631 | 0.7628 | 0.1013 |
| Aa43 | Martínez-Cruz et al. 2002 | 8 | 0.5101 | 0.5164 | 0.0265 |
| IEAAAG09 | Busch et al. 2005 | 4 | 0.5565 | 0.5548 | 0.0580 |
| IEAAAG11 | Busch et al. 2005 | 5 | 0.6815 | 0.6918 | 0.2591 |
| Hal04 | Hailer et al. 2005 | 5 | 0.7482 | 0.7291 | 0.0778 |
| Hal10 | Hailer et al. 2005 | 5 | 0.6415 | 0.6102 | 0.3394 |

Table S2. Apparent survival probabilities ($\phi$) of breeding eastern imperial eagles in East Hungary estimated from the unconstrained time model {$\phi$(sex $\times$ time), p(sex $\times$ time)}. Estimates for the last interval of the study (2021–2022) are not provided since they cannot be estimated separately from encounter probabilities in models where both survival and encounter are time-dependent.

| **Parameter** | **Sex** | **Estimate (ϕ)** | **SE (ϕ)** | **95% CI (ϕ)** |
| --- | --- | --- | --- | --- |
| $\phi$_2011–2012_ | Male | 0.7802 | 0.1147 | 0.4890 – 0.9294 |
|  | Female | 0.9403 | 0.0546 | 0.7009 – 0.9906 |
| $\phi$_2012–2013_ | Male | 0.8066 | 0.1002 | 0.5420 – 0.9363 |
|  | Female | 0.8665 | 0.0532 | 0.7249 – 0.9412 |
| $\phi$_2013–2014_ | Male | 0.8826 | 0.0824 | 0.6126 – 0.9728 |
|  | Female | 0.9554 | 0.0394 | 0.7779 – 0.9924 |
| $\phi$_2014–2015_ | Male | 1.0000 | 0.0000 | 1.0000 – 1.0000 |
|  | Female | 0.8910 | 0.0407 | 0.7825 – 0.9490 |
| $\phi$_2015–2016_ | Male | 0.9101 | 0.0791 | 0.6036 – 0.9854 |
|  | Female | 0.9658 | 0.0348 | 0.7819 – 0.9955 |
| $\phi$_2016–2017_ | Male | 1.0000 | 0.0000 | 1.0000 – 1.0000 |
|  | Female | 0.8937 | 0.0401 | 0.7863 – 0.9505 |
| $\phi$_2017–2018_ | Male | 0.7716 | 0.1186 | 0.4745 – 0.9267 |
|  | Female | 0.8539 | 0.0463 | 0.7384 – 0.9237 |
| $\phi$_2018–2019_ | Male | 1.0000 | 0.0000 | 1.0000 – 1.0000 |
|  | Female | 0.9671 | 0.0616 | 0.3971 – 0.9992 |
| $\phi$_2019–2020_ | Male | 1.0000 | 0.0000 | 1.0000 – 1.0000 |
|  | Female | 0.9175 | 0.0697 | 0.6466 – 0.9854 |
| $\phi$_2020–2021_ | Male | 1.0000 | 0.0002 | 0.9996 – 1.0000 |
|  | Female | 1.0000 | 0.0003 | 0.9995 – 1.0000 |
